# *Achrysocharoides* Girault (Hymenoptera, Eulophidae) new to tropical America, with eight new species

**DOI:** 10.3897/zookeys.173.2653

**Published:** 2012-03-02

**Authors:** Christer Hansson

**Affiliations:** 1 Scientific Associate of the Entomology Department, the Natural History Museum, London SW7 5BD, United Kingdom

**Keywords:** Neotropical, leafminer parasitoids, Gracillariidae, *Gliricidia sepium*, identification key, Chalcidoidea, Entedoninae, *Kratoysma*, junior synonym, recombination

## Abstract

The genus *Achrysocharoides* Girault is here reported for the first time from tropical America. Included are ten species, eight newly described: *Achrysocharoides asperulus*, *Achrysocharoides callisetosus*, *Achrysocharoides cuspidatus*, *Achrysocharoides foveatus*, *Achrysocharoides infuscus*, *Achrysocharoides mediocarinatus*, *Achrysocharoides purpureus*, *Achrysocharoides sulcatus*, and two already known: *Achrysocharoides ecuadorensis* (Hansson) and *Achrysocharoides gliricidiae* (Hansson & Cave). All species are included in an identification key, diagnosed, described and illustrated. Only one of the species, *Achrysocharoides gliricidiae,* has a host record, an endoparasitoid in a leafmining Gracillariidae (Lepidoptera) on *Gliricidia sepium* (Fabaceae), thus conforming to the biology of extralimital *Achrysocharoides* species. The genus *Kratoysma* Bouček is here established as a junior synonym of *Achrysocharoides*, and the following species previously in *Kratoysma* are here recombined to *Achrysocharoides*: *Kratoysma citri* Bouček, *Kratoysma ecuadorensis* Hansson, *Kratoysma gliricidiae* Hansson & Cave, *Kratoysma longifacies* Hansson, *Kratoysma nepalensis* Hansson, *Derostenus usticrus* Erdös.

## Introduction

Species of *Achrysocharoides* Girault are unusually host/host plant specific (e.g. Askew and Ruse 1974). They develop as koinobiont endoparasitoids on larvae of leafmining microlepidoptera, mainly on species in the genus *Phyllonorycter* Hübner (Lepido- ptera: Gracillariidae) (e.g. Bryan 1980a). They have species-specific, and diverse, sex allocation strategies (West et al. 1999), courtship behaviour (Bryan 1980b), and sex-specific size differences (Askew and Ruse 1974). Furthermore, Hansson et al. (ms submitted) demonstrated that species of *Achrysocharoides* co-occurring simultaneously on the same plant, i.e. sympatric species, showed reproductive character displacement of newly discovered visual signaling attributes, i.e. strongly indicating them as devices for reproductive isolation. These visual signals featured wing interference patterns (WIPs), a new type of character recently discovered by Shevtsova et al. (2011). Thus *Achryso- charoides* possesses physical attributes and behaviours that make it very interesting as a model group for evolutionary studies.

Knowledge of *Achrysocharoides* is almost exclusively confined to the temperate parts of the Northern Hemisphere (Shevtsova and Hansson 2011, and references therein), with 22 species known from Europe (now 23 with the recombined *Achrysocharoides usticrus* (Erdös)), 22 from North America and 10 from Japan. At least nominally no species were previously recorded from the Neotropical region. However, with the synonymization of *Kratoysma* with *Achrysocharoides*, in this paper, two species are actually known from tropical America. One of these species is with a host record and similar to northern temperate species it is an endoparasitoid in leafmining Gracillariidae. With the addition of the eight new species described here from tropical America the basic knowledge of this biologically interesting group has increased considerably, opening up the possibility of further research of the evolution of this group in a tropical area.

## Morphological abbreviations and acronyms

**HE** = height of eye; **HW** = height of forewing; **LG** = length of gaster; **LM** = length of marginal vein; **LW** = length of forewing, measured from base of marginal vein to apex of wing; **MM** = length of mesosoma; **MS** = malar space; **OOL** = distance between one posterior ocellus and eye; **PM** = length of postmarginal vein; **POL** = distance between posterior ocelli; **POO** = distance between posterior ocelli and occipital margin; **ST** = length of stigmal vein; **WH** = width of head; **WM** = width of mouth; **WT** = width of thorax. For illustrations of the morphological terms see www.neotropicaleulophidae.com.

Collection acronyms used are: **BMNH** = Natural History Museum, London, England; **CH** = collection of Christer Hansson; **CNC** = Canadian National Collection of Insects, Ottawa, Canada; **INBio** = Instituto Nacional de Biodiversidad, Santo Domingo, Costa Rica; **LUZM** = Lund University Zoological Museum, Lund, Sweden; **MIUCR** = Museo de Insectos, Universidad de Costa Rica, San Pedro, Costa Rica; **TAMU** = Texas A&M University, College Station, U.S.A; **USNM** = United States National Museum of Natural History, Washington, D.C., U.S.A.

The ratios, summarized in Table 1, are based on the holotype and one of the paratypes (if present) of the other sex.

**Table 1.** Ratios, for an explanation of the morphological abbreviations see above.

	**HE/MS/WM**	**POL/OOL/POO**	**WH/WT**	**LW/LM/HW**	**PM/ST**	**MM/LG**
*Achrysocharoides asperulus* sp. n., female	3.0/1.0/1.4	1.1/1.0/1.2	1.1	1.6/1.0/1.1	1.0	1.1
*Achrysocharoides callisetosus* sp. n., female	2.4/1.0/1.1	3.6/2.2/1.0	1.2	1.6/1.0/1.1	0.6	1.1
*Achrysocharoides cuspidatus* sp. n., female	3.2/1.0/1.6	1.8/1.0/1.3	1.3	1.7/1.0/1.1	1.0	0.9
*Achrysocharoides ecuadorensis*(Hansson), female	2.8/1.0/1.5	3.5/2.7/1.0	1.2	1.7/1.0/1.2	1.1	0.9
*Achrysocharoides foveatus* sp. n., female	3.3/1.0/1.1	2.6/1.6/1.0	1.2	1.7/1.0/1.1	1.0	1.0
*Achrysocharoides gliricidiae*(Hansson & Cave),female	3.1/1.0/1.1	1.6/1.1/1.0	1.2	1.5/1.0/1.0	1.3	1.1
*Achrysocharoides gliricidiae*(Hansson & Cave),male	3.0/1.6/1.0					1.2
*Achrysocharoides infuscus* sp. n., female	2.7/1.0/1.7	2.0/1.1/1.0	1.2	1.7/1.0/1.1	1.0	0.9
*Achrysocharoides infuscus* sp. n., male	2.2/1.0/1.3					1.0
*Achrysocharoides medio-carinatus* sp. n.,female	3.6/1.0/2.0	1.5/1.0/1.0	1.1	1.6/1.0/1.0	1.5	1.0
*Achrysocharoides purpureus* sp. n., female	3.2/1.0/1.6	2.4/1.8/1.0	1.3	1.8/1.0/1.1	0.8	1.0
*Achrysocharoides sulcatus* sp. n., female	3.2/1.0/1.5	3.3/1.8/1.0	1.1	1.5/1.0/1.0	0.9	0.7–1.0

**Figure 1.**
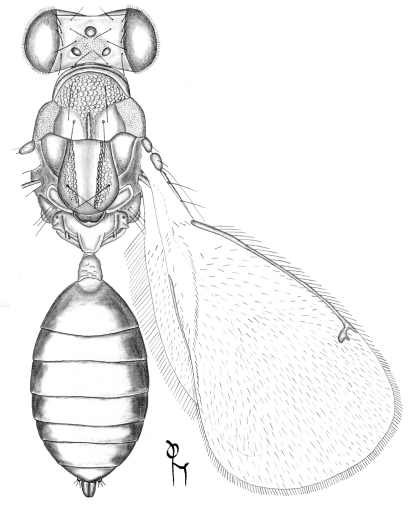
*Achrysocharoides gliricidiae* (Hansson & Cave), female.

**Figure 2.**
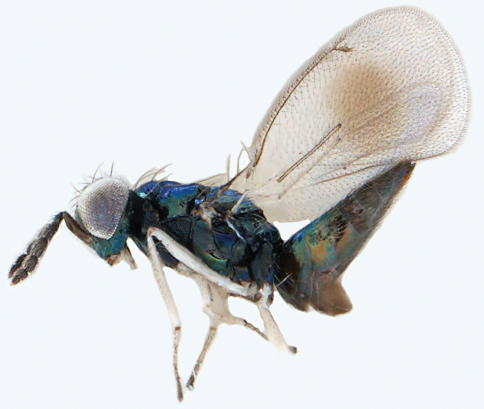
*Achrysocharoides infuscus* sp. n., female.

## Results and discussion

The analysis, based on external morphological characters, of the material at hand resulted in ten species – two already described and eight undescribed species. Wing interference patterns (WIPs) was one character-set included in the analysis. However, these patterns had no useful information for species separation as they were very similar between species (as in Fig. 26). Hansson et al. (manuscript, submitted) called this pattern ancestral and it was present in all allopatric species of *Achrysocharoides* in Europe. This pattern is with a narrow band in the forewing, from the stigmal vein to the posterior margin of the wing, with a thick membrane inside and a thin membrane outside the band. Applying the results from Hansson et al., the prediction is that all species included here are allopatric.

Nine of the species included here belong to the *gahani* species-group, characterized by having an edge along occipital margin, a transverse carina close to posterior margin of dorsal pronotum, two submedian carinae on propodeum, and with a row of foveae laterally on scutellum. *Achrysocharoides gliricidiae* lacks propodeal median carinae (Fig. 31), and *Achrysocharoides mediocarinatus* has just a single median carina (Fig. 42). However, both species have the other characters for the group, and they are thus best placed in the *gahani*-group. The placement of *Achrysocharoides foveatus* into a species-group is problematic, and it is left as unplaced. This species has a sharp edge along the occipital margin (Fig. 56) and foveae on lateral part of scutellum (Fig. 59), but lacks a pronotal carina and longi- tudinal carinae on propodeum (Fig. 57). Apart from the *gahani*-group three other species-groups may have pits on the scutellum (Kamijo 1991): *titiani*-, *clypeatus*-, and *latreilleii*-groups, but none of these groups have a carinate occipital margin, submedian carinae on propodeum, or – except some species in the *titiani*-group – a transverse carina on pronotum.

When describing *Kratoysma* Bouček (1965) compared it with subgenus *Kratochviliana* Malač (of genus *Chrysocharis* Förster), with *Enaysma* Delucchi (now a synonym of *Achrysocharoides*) – hence the name *Krato*(chviliana)–(Ena)*ysma* – and with *Pediobius* Walker. He found the “general aspect” of *Kratoysma* to be like that of *Kratochviliana*, the frons with a raised cross-line (= raised frons above frontal suture) was shared with *Enaysma*, while two other characters, wing venation and presence of lateral plicae on the propodeum, were shared with *Pediobius*. Some of the features mentioned by Bouček are hard to define: “general aspect” and unspecified “wing venation” are open to any interpretation. The raised frontal suture and the presence of propodeal plicae are more definite features. The raised frons above frontal suture (in the female) is an apomorphy also for *Achrysocharoides*, while the propodeal plicae are present in several other genera of Entedoninae, e.g. *Pediobius* and in some species of *Achrysocharoides*. *Achrysocharoides* has another apomorphy unknown to Bouček at the time of the description of *Kratoysma*: a longitudinal carina on the lateral downsloping part of pronotum (Gumovsky 2007) (Figs 60–63). This longi- tudinal carina is also present in *Kratoysma* *usticrus*, the type species for *Kratoysma* (Fig. 61). Hence there are two unique apomorphies for *Achrysocharoides*+*Kratoysma,* the raised frontal suture and the longitudinal carina on lateral pronotum. The diffe- rences between these genera at the time when *Kratoysma* was described were mainly two: presence in *Kratoysma* of a transverse pronotal carina and propodeal plicae, with the absence of both in *Achrysocharoides*. However, the addition of new species of *Achrysocharoides* from Japan (Kamijo 1990a, 1990b) and North America (Kamijo 1991) has demonstrated a variation in both characters, a variation that obscures the borderline, based on morphological characters, between these two genera and which presents major difficulties for the definition of them. Hence there are no longer any derived characters to keep *Achrysocharoides* and *Kratoysma* as separate genera, and *Kratoysma*, being the junior name, is hereby synonymizedwith *Achrysocharoides*. The following species, previously placed in *Kratoysma,* are hereby transferred to *Achryso- charoides*: *A*. *citri* (Bouček), comb. n., *Achrysocharoides ecuadorensis* (Hansson), comb. n., *Achrysocharoides gliricidiae* (Hansson & Cave), comb. n., *Achrysocharoides longifacies* (Hansson), comb. n., *Achrysocharoides nepalensis* (Hansson), comb. n., *Achrysocharoides usticrus* (Erdös), comb. n.

### Achrysocharoides

Girault

http://species-id.net/wiki/Achrysocharoides

Achrysocharoides Girault, 1913:72. Type species: *Chrysocharis sarcophagus* Girault, by original designation.
Neoderostenus Girault, 1915:180. Type species *Neoderostenus australiensis* Girault, 1915:180, by original designation. Synonymized by Peck (1951).
Enaysma Delucchi, 1954:1. Type species: *Enaysma zwoelferi* Delucchi, by orginal designation. Synonymized by Yoshimoto (1977).
Kratoysma Bouček, 1965:5–6. Type species: *Derostenus usticrus* Erdös, by original designation. Syn. n.

#### The classification into species-groups.

The subdivision of *Achrysocharoides* was initiated by Graham (1959) who divided the European species into two subgenera, *Enaysma* Delucchi and *Pentenaysma* Graham. These correspond with the two species-groups, *atys*- and *latreilleii*-groups, which Bryan (1980a) introduced for the European species, thus abandoning the formal subdivision into subgenera. Yoshimoto (1977) divided the Nearctic species into two species-groups, the *gahani*- and *guizoti*-groups. Kamijo (1991) transferred some of the Nearctic species from the *guizoti*-group to either of the two newly erected *clypeatus*- and *titiani*-groups, and removed the remaining species in the *guizoti*-group to the *latreilleii*-group, thus terminating the *guizoti*-group. Kamijo (1990b) established the *crassinervis*-group for two species from Japan and one undescribed species from Nepal. Hence there are currently six species-groups in *Achrysocharoides*: *atys*-, *clypeatus*-, *crassinervis*-, *gahani*-, *latreilleii*-, and *titiani*-groups. See Kamijo (1991) for group-diagnostic characters.

#### Diagnosis.

**Figures 3–7.**
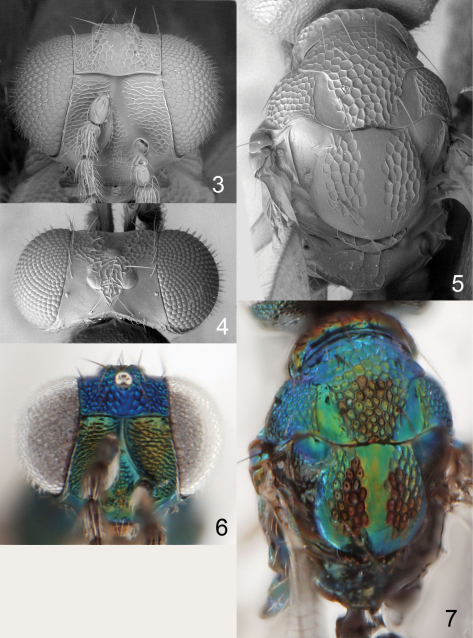
*Achrysocharoides asperulus* sp. n., female. **3** Head, frontal **4** Vertex **5** Thoracic dorsum **6** Head, frontal **7** Thoracic dorsum.

Eyes densely pubescent (e.g. Fig. 3); females with frontal suture as a raised carina (i.e. with frons just above frontal suture protruding), straight (e.g. Fig. 3); males with frontal suture straight to slightly V-shaped, sometimes missing; females with antennal scrobes indistinct (i.e. not as narrow grooves) joining below frontal suture (e.g. Fig. 3); lateral downsloping part of pronotum with a longitudinal carina (Figs 60–63); postmarginal vein short, 0.5–1.5× as long as stigmal vein. *Achrysocharoides* is similar to *Apleurotropis*, but has a short postmarginal vein (in *Apleurotropis* postmarginal vein is 2.8–3.7× as long as the stigmal vein), with antennal scrobes in female joining below frontal suture (antennal scrobes join the frontal suture separately in *Apleurotropis*).

#### Identification.

To separate *Achrysocharoides* from other Eulophidae genera the keys in Bouček (1988) (Australasia), Gibson et al. (1997) (Nearctic), Graham (1959) (Europe) are useful. To differentiate *Achrysocharoides* from other genera of Entedon- inae in tropical America the matrix key on the website http://www.neotropicaleulophidae.com can be used.

#### Description.

Female flagellum with a 2-segmented clava, in male with a 2-segmented clava or with all 5 flagellomeres distinctly separated; male flagellomeres with scattered setae; male scape enlarged, frequently with a species-specific shape, ventral sensory area present along entire scape; sensilla ampullacea globular, symmetric (type I sensu Hansson (1990)), present on all flagellomeres. Antenna with discoid anelli. Mandibles with two large teeth at apex, with one or several smaller teeth above large teeth. Clypeus not delimited. Malar sulcus present. Males with a more or less developed cross-ridge below antennal toruli. Frons occasionally with an indistinct groove between median ocellus and frontal suture. Female with frons just above frontal suture protruding, hence frontal suture appear to be a raised carina. Frontal suture in female straight; in male straight to slightly V-shaped, but sometimes missing. Antennal scrobes usually join below frontal suture in females, join on or below frontal suture in males, scrobes absent in males of some species. Occipital margin with raised carina or an edge, or rounded; occiput with a median fold/groove, at least close to occipital margin.

Pronotum with or without a transverse carina. Midlobe of mesoscutum with two pairs of setae, sometimes with an indistinct median groove in posterior ½; notauli more or less distinct in anterior 1/2, in posterior 1/2 present as weakly delimited depressions which are smooth to weakly reticulate. Scutellum with one pair of setae; sometimes with an anteromedian groove; with or without detached lateral foveae or rows of foveae. Transepimeral sulcus almost straight to weakly curved. Dorsellum visible in dorsal view. Forewing with costal cell usually wider than width of base of submarginal vein; postmarginal vein 0.5-1.5× as long as stigmal vein, usually about as long as stigmal vein. Propodeum without longitudinal ridges, or with a complete median carina - undivided or branched in posterior half - median carina sometimes incomplete and present only in posterior 1/3, or with two complete submedian carinae that run parallel or diverge weakly to strongly towards posterior part of propodeum.

Petiole 0.5–1.5× as long as wide, smooth and shiny or with some irregular sculpture, sometimes with anterolateral corners, ventral surface smooth. Male genitalia as in most other genera of Entedoninae, i.e. with normal volsellar setae, one parameral setae at the apex of phallobase, with two digital spines (Hansson 1996).

#### Biology.

Endoparasitoids of leafmining Lepidoptera of the family Gracillariidae, mainly the genus *Phyllonorycter* Hübner (Bryan 1980a).

#### Distribution.

Australia (Girault 1913, Bouček 1988), Canada (Kamijo 1991, Miller 1962, Yoshimoto 1977), Japan (Kamijo 1990a, 1990b), Nepal (Hansson 1985), New Zealand (Bouček 1988), Pakistan (Hansson 1985), Papua New Guinea (Bouček 1988), USA (Kamijo 1991), and Europe (many countries, e.g. Bouček and Askew 1968). In tropi- cal America: Costa Rica, Ecuador, Guatemala, Honduras, Mexico, Trinidad and Tobago.

#### Key to the Neotropical species of *Achrysocharoides*

**Table:** 

1	Pronotal collar without transverse carina (Fig. 57)	*Achrysocharoides foveatus* sp. n. (female)
–	Pronotal collar with a transverse carina (e.g. Figs 5, 10, 15)	2
2	Median propodeum smooth, without longitudinal carinae (Figs 25, 31)	*Achrysocharoides gliricidiae* (Hansson & Cave) (female, male)
–	Median propodeum with 1 or 2 longitudinal carinae (Figs 15, 39, 42)	3
3	Propodeum with 1 complete median carina (Fig. 42)	*Achrysocharoides mediocarinatus* sp. n. (female)
–	Propodeum with 2 submedian carinae, diverging towards posterior part of propodeum (Figs 15, 39)	4
4	Entire scutellum with raised and very strong reticulation, with only anteromedian and posteromedian parts smooth (Fig. 47)	*Achrysocharoides purpureus* sp. n. (female)
–	Either with entire median part of scutellum smooth, i.e. with a smooth and complete longitudinal band (Figs 20, 39), *or* scutellum with engraved and weak reticulation (Figs 10, 11)	5
5	Scape (Fig. 12), femora and tibiae dark brown with metallic tinges	*Achrysocharoides callisetosus* sp. n. (female)
–	Scape white to infuscate (dark brown in female *Achrysocharoides infuscus*), femora and tibiae predominantly white, femora occasionally pale brown	6
6	Frons above frontal suture smooth and shiny (Figs 18, 35, 50)	7
–	Frons above frontal suture with raised and strong reticulation (Figs 3, 13, 16)	9
7	Propodeum with plicae (Fig. 20)	*Achrysocharoides ecuadorensis* (Hansson) (female)
–	Propodeum without plicae (Figs 39, 52)	8
8	Female scape dark brown (Fig. 2), mesoscutum golden-green and scutellum golden-red (Fig. 34); posteromedian 2/3 of scutellum smooth (Fig. 39)	*Achrysocharoides infuscus* sp. n. (female, male)
–	Female scape whitish to yellowish-brown (Fig. 53), mesoscutum and scutellum golden-green (Fig. 54); posteromedian 2/3 of scutellum with engraved reticulation (Fig. 54)	*Achrysocharoides sulcatus* sp. n. (female, male)
9	Midlobe of mesoscutum with meshes of reticulation small and with reti- culate part posteriorly narrowing off to a point towards anterior scutellum (Fig. 15)	*Achrysocharoides cuspidatus* sp. n. (female)
–	Midlobe of mesoscutum with meshes of reticulation large and with reticulate part posteriorly wide (Fig. 5)	*Achrysocharoides asperulus* sp. n. (female)

## Species treatments

### Species-group *gahani*

#### Achrysocharoides asperulus sp. n.

urn:lsid:zoobank.org:act:C7FB8FD2-28DE-4FBC-84DE-B1CE213786D5

http://species-id.net/wiki/Achrysocharoides_asperulus

Figures 3–7

##### Material.

Holotype female (INBio) glued to a card, labelled “Costa Rica: Heredia, 16 km SSE La Virgen, 1050–1150 m, 10°16'N, 84°05'W, 9–29.iii.2001, 11/M/NOTN, INBio.OET-ALAS intersect”.

##### Diagnosis..

Scutellum predominantly with raised and very strong reticulation, median 1/5 smooth (Fig. 5); upper frons and vertex inside ocellar triangle with raised and very strong reticulation and vertex outside ocellar triangle smooth (Figs 3, 4, 6); postmarginal vein 1.0× as long as stigmal vein; median propodeum with two irregular subparallel carinae (Fig. 5); propodeal callus with three setae; pronotum with a transverse carina close to posterior margin (Fig. 5).

##### Description.

FEMALE. Length 1.3 mm.

Scape white with apical ¼ brown, pedicel and flagellum dark brown with metallic tinges. Frons below frontal suture golden-green, above frontal suture metallic bluish-green (Fig. 6). Vertex metallic bluish-purple, golden-red inside ocellar triangle. Mesoscutum, scutellum and propodeum metallic bluish-green with red tinges (Fig. 7). Fore coxa white, mid and hind coxae dark and metallic; femora, tibiae and tarsi white, ventral part of fore and mid femora, dorsal part of hind femur, and basal ½ of mid tibia pale brown. Forewing hyaline. Petiole black with metallic purple tinges. Gastral tergites 1+2 metallic bluish-green, remaining tergites metallic dark purple.

Frons with raised and strong reticulation (Fig. 3). Vertex smooth, inside ocellar triangle with raised and strong reticulation (Fig. 4). Occipital margin with sharp carina behind ocellar triangle (Fig. 4). Ratios: length of flagellomeres I/II/III/IV/V (excl. spicule) 1.0/1.2/1.2/1.0/1.0.

Pronotum with a strong transverse carina close to posterior margin (Fig. 5). Meso- scutum with raised and strong reticulation; notaular depressions smooth and shiny (Fig. 5). Scutellum with raised and strong reticulation, median 1/5 smooth (Fig. 5). Axillae reticulate with anterior 1/3 smooth and shiny (Fig. 5). Dorsellum flat with two foveae anterolaterally (Fig. 5). Forewing speculum closed below. Propodeum medially with two irregular longitudinal carinae which are ±parallel; propodeal callus with three setae (Fig. 5).

Petiole 0.6× as long as wide, with weak sculpture. Gaster oval-shaped.

MALE. Unknown.

##### Etymology.

From the Latin *asper* = rough, in its diminutive form = *asperulus*, referring to the strong reticulation on thoracic dorsum.

##### Distribution.

Costa Rica.

#### Achrysocharoides callisetosus sp. n.

urn:lsid:zoobank.org:act:635C2C5B-0B40-4821-AA42-30F7AEDB4BEA

http://species-id.net/wiki/Achrysocharoides_callisetosus

Figures 8–12

##### Material.

Holotype female (BMNH) glued to a card, labelled “Costa Rica: San José, 19 km S 1km W Empalme, Mirador Quetzales, 2600 m, ii.2000, P. Hanson”. Paratype: 1♀ COSTA RICA. **San José:** Cerro de la Muerte, 6 km S Empalme, 2800 m, 8.ix.1991, P. Hanson (BMNH).

##### Diagnosis.

**Figures 8–12.**
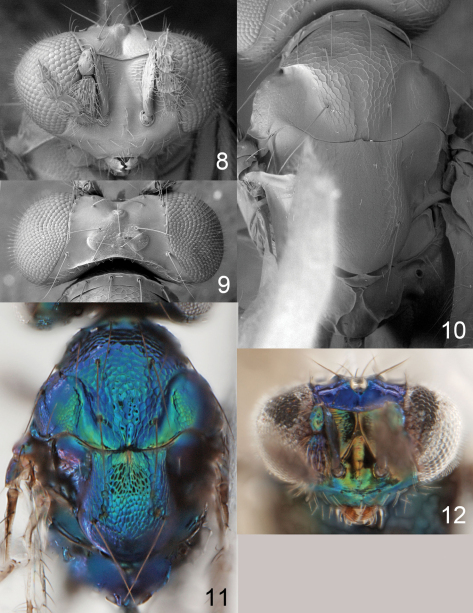
*Achrysocharoides callisetosus* sp. n., female. **8** Head, frontal **9** Vertex **10** Thoracic dorsum **11** Thoracic dorsum **12** Head, frontal.

Scutellum with engraved and weak reticulation and with two sublateral rows of foveae (Fig. 10); postmarginal vein 0.6× as long as stigmal vein; propodeum with two submedian carinae, strongly diverging posteriorly (Fig. 10); propodeal callus with 8 setae; scape, femora and tibiae dark brown with metallic tinges; head transverse, 1.7× as wide as high (Fig. 8); pronotum with a transverse carina close to posterior margin (Fig. 10).

##### Description.

FEMALE. Length 1.7 mm.

Entire antenna dark brown. Frons golden-green (Fig. 12). Vertex metallic bluish-purple, golden-green inside ocellar triangle. Mesoscutum, scutellum and propodeum metallic bluish-green (Fig. 11). Coxae black with metallic purple tinges, femora and tibiae dark brown with metallic tinges; fore tarsus pale brown, mid and hind tarsi with tarsomeres 1+2 white, 3 pale brown, 4 dark brown. Forewing hyaline with an infuscate median spot. Petiole golden-green. Gastral tergites 1+2 metallic bluish-green, remaining tergites metallic dark purple.

Frons below frontal suture with raised and weak reticulation, above frontal suture smooth (Fig. 8). Vertex smooth with weak reticulation inside ocellar triangle (Fig. 9). Occipital margin with sharp carina (Fig. 9). Ratios: length of flagellomeres I/II/III/IV/V (excl. spicule) 2.2/1.8/2.0/1.2/1.0.

Pronotum with a strong transverse carina close to posterior margin (Fig. 10). Midlobe of mesoscutum with raised and strong reticulation, sidelobes with engraved and weak reticulation; notaular depressions smooth and shiny; midlobe with a median groove in posterior 1/3 (Figs 10, 11). Scutellum with engraved and weak reticulation and with two sublateral rows of foveae (Fig. 10). Axillae smooth and shiny (Fig. 10). Dorsellum flat with two foveae anterolaterally (Fig. 10). Forewing speculum closed below. Propodeum smooth and shiny, with two submedian carinae strongly diverging towards petiolar foramen, and with complete plicae (Fig. 10); propodeal callus with eight setae. Petiolar foramen semicircular.

Petiole 1.5× as long as wide, dorsal surface with weak sculpture. Gaster oval-shaped.

MALE. Unknown.

##### Etymology.

From “propodeal callus” and the Latin *setosus* = bristly, referring to the numerous setae on propodeal callus.

##### Distribution.

Costa Rica. The two available specimens have both been collected at high altitude (2600–2800 m).

#### Achrysocharoides cuspidatus sp. n.

urn:lsid:zoobank.org:act:24ABD9B5-7FC7-4622-BE03-72F53B53B8DB

http://species-id.net/wiki/Achrysocharoides_cuspidatus

Figures 13–17

##### Material.

Holotype female (INBio) glued to a card, labelled “Costa Rica: Punta– renas, 1km S del Cerro Biolley, 1300–1450m, 23.viii–13.ix.1996, R. Villalobos, Malaise Trap, LS 331700/572100, #44870”.

**Figures 13–17.**
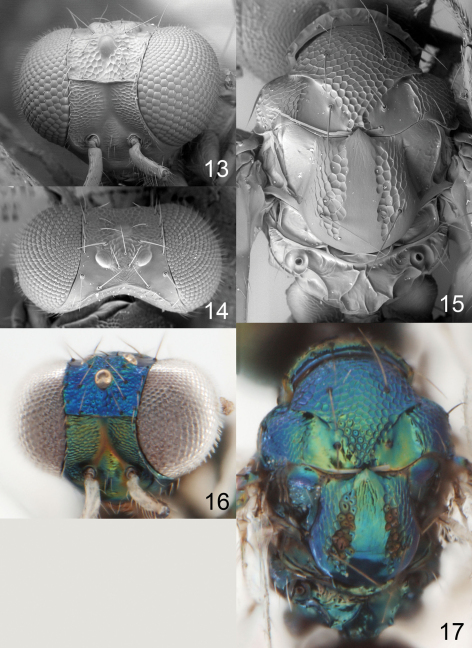
*Achrysocharoides cuspidatus* sp. n., female. **13** Head, frontal **14** Vertex **15** Thoracic dorsum **16** Head, frontal **17** Thoracic dorsum.

**Diagnosis**. Frons above frontal suture with raised and strong reticulation (Fig. 13); pronotum with a transverse carina close to posterior margin (Fig. 15); midlobe of mesoscutum with raised and strong reticulation, reticulate part triangular in posterior part with narrow part pointing at scutellum (Fig. 15); scutellum with two sublateral rows of foveae, surface between rows of foveae smooth and shiny (Fig. 15); postmarginal vein 1.0× as long as stigmal vein; propodeum with two submedian carinae, strongly diverging posteriorly, and with plicae (Fig. 15); propodeal callus with three setae.

##### Description.

FEMALE. Length 1.6 mm.

Scape white, pedicel metallic bluish-green, flagellum dark brown. Frons below frontal suture golden-green with scrobes golden-red, above frontal suture metallic bluish-purple (Fig. 16). Vertex metallic bluish-green. Mesoscutum, scutellum and propodeum metallic bluish-green (Fig. 17). Fore coxa white with base infuscate, mid coxa pale brown with metallic tinges, hind coxa dark and metallic; remaining parts of legs white. Forewing hyaline with a weak median infuscate spot. Petiole dark brown with metallic tinges. Gaster with tergites 1+2 metallic bluish-green, remaining tergites brown with metallic blue tinges.

Frons with raised and strong reticulation, scrobes smooth (Fig. 13). Vertex smooth and shiny, inside ocellar triangle with raised and strong reticulation (Fig. 14). Occipital margin with a sharp carina behind ocellar triangle (Fig. 14). Ratios: length of flagellomeres I/II/III/IV/V (excl. spicule) 1.7/1.7/1.6/1.0/1.0.

Pronotum with a strong transverse carina close to posterior margin (Fig. 15). Midlobe of mesoscutum with raised and strong reticulation; sidelobes with raised and weak reticulation; notaular depressions smooth and shiny (Fig. 15). Scutellum with two sublateral rows of foveae, medially between rows of foveae smooth and shiny, outside rows of foveae with raised and weak reticulation (Fig. 15). Axillae smooth and shiny (Fig. 15). Dorsellum slightly concave, smooth and shiny, with two foveae anterolaterally (Fig. 15). Forewing speculum closed below; costal cell bare. Propodeum with two submedian carinae, strongly diverging posteriorly, and with plicae; propodeal surface smooth and shiny (Fig. 15); propodeal callus with three setae. Petiolar foramen rounded.

Petiole as long as wide, dorsal surface smooth. Gaster slightly elongate.

MALE. Unknown.

##### Etymology.

From the Latin *cuspidatus* = make pointed, referring to reticulate part on midlobe of mesoscutum that is pointed towards scutellum.

##### Distribution.

Costa Rica.

#### Achrysocharoides ecuadorensis

(Hansson) comb. n.

http://species-id.net/wiki/Achrysocharoides_ecuadorensis

Figures 18–22 63

Kratoysma ecuadorensis Hansson & Cave, 1993:256.

##### Diagnosis.

Pronotum with a transverse carina close to posterior margin (Fig. 20); scutellum predominantly smooth and shiny, with two sublateral rows of foveae (Fig. 20); propodeum with plicae and two submedian carinae (Fig. 20).

##### Description.

See Hansson & Cave (1993).

##### Distribution.

Ecuador (Hansson & Cave 1993).

**Figures 18–22.**
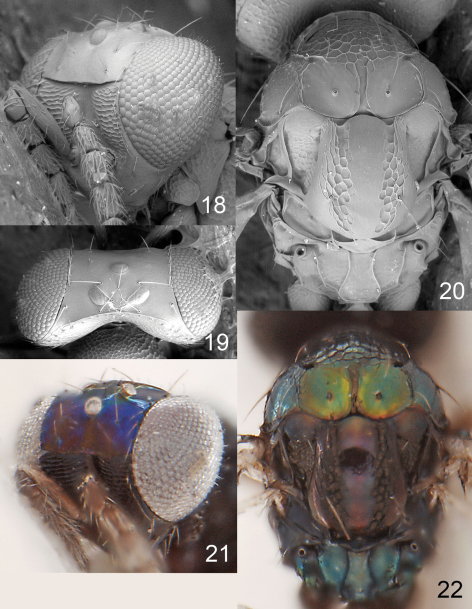
*Achrysocharoides ecuadorensis* (Hansson), female. **18** Head, frontal **19** Vertex **20** Thoracic dorsum **21** Head, frontal **22** Thoracic dorsum.

#### Achrysocharoides gliricidiae

(Hansson & Cave) comb.n.

http://species-id.net/wiki/Achrysocharoides_gliricidiae

Figures 1 23 30

Kratoysma gliricidiae Hansson & Cave, 1993:254.

##### Diagnosis.

Pronotum with a transverse carina close to posterior margin (Fig. 31); scutellum smooth with two sublateral rows of foveae (Fig. 31); propodeum without longitudinal carinae medially (Fig. 31); propodeal callus with three setae.

##### Description.

See Hansson & Cave (1993).

##### Distribution.

Costa Rica (**new record**, 40♀ 4♂, BMNH, CH, CNC, INBio, MIUCR, USNM), Honduras (Hansson & Cave 1993), Mexico (**new record**, 2♀, TAMU), Trinidad & Tobago (**new record**, 1♀, CNC), USA (Arizona) (**new record**, 1♀, CNC).

##### Biology.

Endoparasitoid of an unidentified Gracillariidae (Lepidoptera) on *Gliricidia sepium* (Fabaceae) (Hansson & Cave 1993).

**Figures 23–26.**
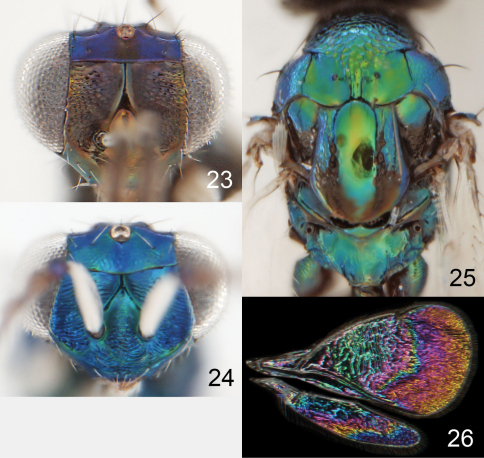
*Achrysocharoides gliricidiae* (Hansson & Cave). **23** Head, frontal, female **24** Head, frontal, male **25** Thoracic dorsum, female **26** Wings, showing wing interference patterns (WIPs).

**Figures 27–31.**
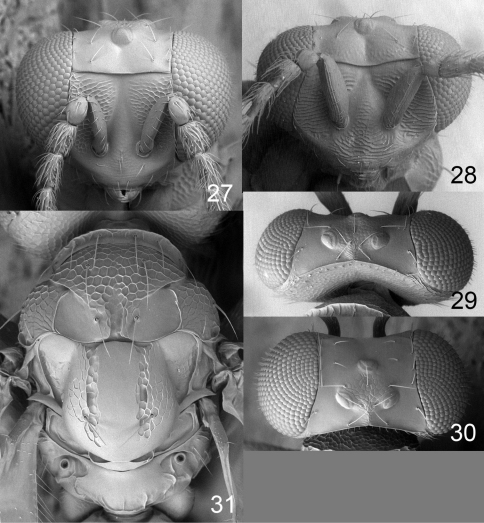
*Achrysocharoides gliricidiae* (Hansson & Cave). **27** Head, frontal, female **28** Head, frontal, male **29** Vertex, male **30** Vertex, female **31** Thoracic dorsum, female.

#### Achrysocharoides infuscus sp. n.

urn:lsid:zoobank.org:act:2136BF7E-A431-495A-BA76-D0280038829B

http://species-id.net/wiki/Achrysocharoides_infuscus

Figures 2 32 39 62

##### Material.

Holotype female (INBio) glued to a card, labelled “Costa Rica: Punta- renas, Estación Altamira, 1450m, 9°02'N, 83°00'W, 7.ii–5.iii.2002, C. Hansson & Parataxonomos”. Paratypes: 3♀ 2♂: COSTA RICA. **Puntarenas**: 3♀ with same label data as holotype (BMNH, INBio). HONDURAS. **Cortés:** 2♂ Parque Nacional San Cusuco, 5km N Buenos Aires, 15°29'N, 83°13'W, 8.iii.1997, C. Hansson (BMNH).

##### Diagnosis.

Female scape dark brown (Fig. 2); pronotum with a transverse carina close to posterior margin (Fig. 39); midlobe of mesoscutum with sidelobes smooth (Fig. 39); scutellum with sublateral parts with raised and strong reticulation, median, lateral and posterior parts smooth (Fig. 39); postmarginal vein 1.0× as long as stigmal vein; propodeum with two submedian carinae, strongly diverging posteriorly (Fig. 39); propodeal callus with three setae.

##### Description.

FEMALE. Length 1.4–1.5 mm.

**Figures 32–34.**
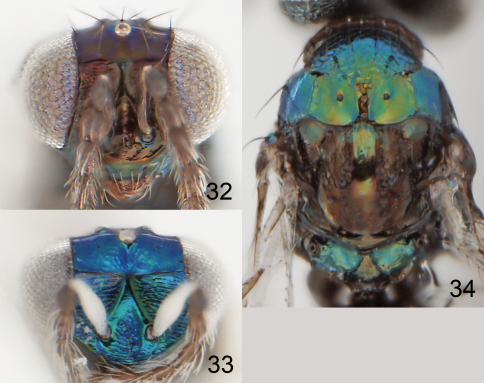
*Achrysocharoides infuscus* sp. n. **32** Head, frontal, female **33** Head, frontal, male **34** Thoracic dorsum.

Antenna dark brown (Fig. 2). Frons below frontal suture golden-red with scrobes golden-green, above frontal suture metallic purple (Fig. 32). Vertex golden-green. Mesoscutum and propodeum metallic bluish-green (Fig. 34). Scutellum golden-red (Fig. 34). Coxae dark brown with metallic tinges; femora pale brown; tibiae and tarsi white. Forewing hyaline with a weak median infuscate spot. Petiole dark brown with metallic tinges. Gaster with tergites 1+2 metallic bluish-green, remaining tergites metallic dark purple.

**Figures 35–39.**
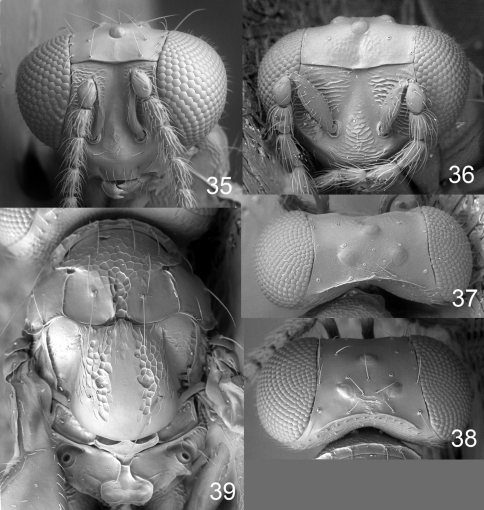
*Achrysocharoides infuscus* sp. n. **35** Head, frontal, female **36** Head, frontal, male **37** Vertex, male **38** Vertex, female **39** Thoracic dorsum, female.

Frons below frontal suture with parts between scrobes and eyes with raised and strong reticulation, remaining parts smooth, above frontal suture smooth (Fig. 35). Vertex smooth and shiny (Fig. 38). Occipital margin with a sharp carina behind ocellar triangle (Fig. 38). Ratios: length of flagellomeres I/II/III/IV/V (excl. spicule) 1.3/1.4/1.3/1.0/1.1.

Pronotum with a strong transverse carina close to posterior margin (Fig. 39). Midlobe of mesoscutum with raised and strong reticulation; sidelobes and notaular depressions smooth and shiny (Fig. 39). Scutellum with sublateral parts with raised and strong reticulation, median, lateral and posterior parts smooth (Fig. 3). Axillae smooth and shiny (Fig. 39). Dorsellum slightly concave, smooth and shiny, with two foveae anterolaterally (Fig. 39). Forewing speculum closed below; costal cell bare. Propodeum with two submedian carinae, strongly diverging posteriorly (Fig. 39); propodeal surface smooth and shiny; propodeal callus with three setae. Petiolar foramen rounded.

Petiole as long as wide, dorsal surface with weak irregular sulpture. Gaster slightly elongate.

MALE. Length 1.2 mm.

Scape (Fig. 33) and femora white. Frons metallic bluish-green (Fig. 33). Gaster with a round white spot in anteromedian 1/3. Colour otherwise as in female.

Frons with interscrobal area with raised and strong reticulation (Fig. 36). Head otherwise as in female.

Mesosoma as in female.

##### Etymology.

From the Latin *infuscus* = dark brown, referring to dark brown scape.

##### Distribution.

Costa Rica and Honduras.

#### Achrysocharoides mediocarinatus sp. n.

urn:lsid:zoobank.org:act:6217EA73-F510-44D7-949A-8B3148494355

http://species-id.net/wiki/Achrysocharoides_mediocarinatus

Figures 40–44

##### Material.

Holotype female (TAMU) glued to a card, labelled “Mexico: Chiapas, San Cristobal, Reserva Huitepec, 7700–7850’, 3.viii.1990, J.B. Woolley, 90/015B”.

##### Diagnosis.

Pronotum with a transverse carina close to posterior margin (Fig. 42); scutellum with two sublateral rows of strong reticulation, remaining surface smooth and shiny (Fig. 42); postmarginal vein 1.5× as long as stigmal vein; propodeum with a complete median carina (Fig. 42); propodeal callus with four setae; coxae white, base of hind coxa dark and metallic.

##### Description.

FEMALE**.** Length 1.8 mm.

Scape white with dorsoapical tip infuscate, pedicel golden-green, flagellum dark brown. Frons below frontal suture golden-green, above metallic bluish-purple (Fig. 43). Vertex metallic bluish-purple, golden-green in posterior part. Mesoscutum, scutellum and propodeum metallic bluish-green (Fig. 44). Legs white, hind coxa dark and metallic at base. Forewing hyaline with a weak median infuscate spot. Petiole dark brown. Gastral tergites 1+2 metallic bluish-green, remaining tergites metallic dark purple.

**Figures 40–44.**
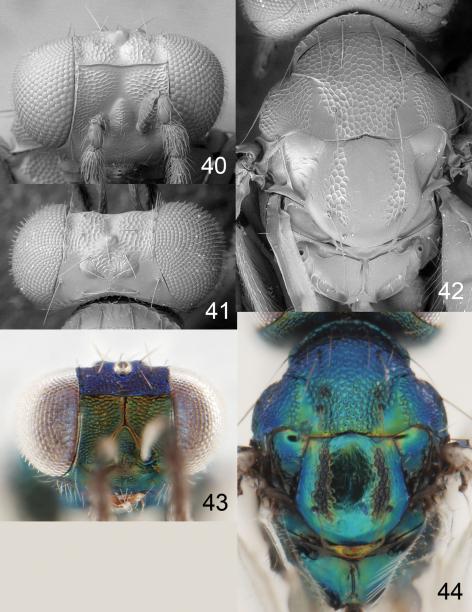
*Achrysocharoides mediocarinatus* sp. n., female. **40** Head, frontal **41** Vertex **42** Thoracic dorsum **43** Head, frontal **44** Thoracic dorsum.

Frons with raised and strong reticulation (Fig. 40). Vertex smooth with raised and strong reticulation inside ocellar triangle (Fig. 41). Occipital margin with sharp carina (Fig. 41). Ratios: length of flagellomeres I/II/III/IV/V (excl. spicule) 1.7/2.0/1.8/1.1/1.0.

Pronotum with a strong transverse carina close to posterior margin (Fig. 42). Meso- scutum with raised and strong reticulation; notaular depressions smooth and shiny (Fig. 42). Scutellum with two sublateral rows of strong reticulation, remaining surface smooth and shiny (Fig. 42). Axillae smooth and shiny (Fig. 42). Dorsellum slightly concave, smooth and shiny, with two foveae anterolaterally (Fig. 42). Forewing speculum closed below. Propodeum smooth and shiny, with a complete median carina (Fig. 42); propodeal callus with four setae. Petiolar foramen semicircular.

Petiole transverse, 0.6× as long as wide, smooth and shiny. Gaster oval-shaped.

MALE. Unknown.

##### Etymology.

From the Latin *medius* = middle, and *carina* = keel, referring to median carina on propodeum.

##### Distribution.

Mexico.

#### Achrysocharoides purpureus sp. n.

urn:lsid:zoobank.org:act:9D6908B8-C723-452B-8C47-35BD90B364B0

http://species-id.net/wiki/Achrysocharoides_purpureus

Figures 45–49

##### Material.

Holotype female (LUZM) glued to a card, labelled “Guatemala: 5 km E Antigua Guatemala, 1780 m, 4.xi.1991, R. Baranowski”.

##### Diagnosis.

Pronotum with a transverse carina close to posterior margin (Fig. 47); scutellum metallic purple with raised and very strong reticulation, without sublateral rows of foveae or meshes (Figs 47, 49); postmarginal vein 0.8× as long as stigmal vein; propodeum with two subparallel carinae, strongly diverging posteriorly (Fig. 47); propodeal callus with three setae.

##### Description.

FEMALE. Length 1.4 mm.

Scape white, pedicel and flagellomeres dark brown with metallic tinges. Frons below frontal suture golden-red, above metallic bluish-purple (Fig. 48). Vertex metallic bluish-purple, golden-green in posterior 1/2. Midlobe of mesoscutum golden-red, sidelobes metallic purple (Fig. 49). Scutellum metallic purple (Fig. 49). Propodeum golden-green (Fig. 49). Coxae dark brown with metallic tinges, fore and mid femora+tibiae+tarsi white, hind femur pale brown, hind tibia and tarsus yellowish-brown. Forewing hyaline with a very weak infuscate median spot. Petiole dark brown with metallic tinges. Gastral tergite 1 metallic bluish-green, remaining tergites metallic dark purple.

**Figures 45–49.**
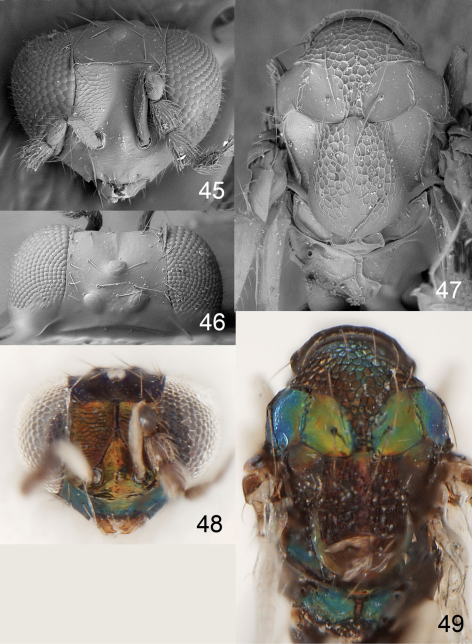
*Achrysocharoides purpureus* sp. n., female. **45** Head, frontal **46** Vertex **47** Thoracic dorsum **48** Head, frontal **49** Thoracic dorsum.

Frons with raised and strong reticulation (Fig. 45). Vertex smooth with engraved and very weak reticulation inside ocellar triangle (Fig. 46). Occipital margin with sharp carina behind ocellar triangle (Fig. 46). Ratios: length of flagellomeres I/II/III/IV/V (excl. spicule) 1.9/1.9/1.6/1.4/1.0.

Pronotum with a strong transverse carina close to posterior margin (Fig. 47). Midlobe of mesoscutum with raised and very strong reticulation, sidelobes with engraved and weak reticulation (Fig. 47); notaular depressions smooth and shiny; midlobe with a weak median groove in posterior ¼. Scutellum with raised and very strong reticulation (Fig. 47). Axillae smooth and shiny (Fig. 47). Dorsellum flat with two foveae anterolaterally (Fig. 47). Forewing speculum closed below. Propodeum with two subparallel carinae, strongly diverging posteriorly (Fig. 47); propodeal callus with three setae. Petiolar foramen semicircular.

Petiole 1.0× as long as wide. Gaster oval-shaped.

Male. Unknown.

##### Etymology.

From the Latin *purpureus* = purple, referring to purple scutellum.

##### Distribution.

Guatemala.

#### Achrysocharoides sulcatus sp. n.

urn:lsid:zoobank.org:act:18EF48BD-BFAC-4930-92F4-64566BBE2D97

http://species-id.net/wiki/Achrysocharoides_sulcatus

Figures 50–54

##### Material.

Holotype female (TAMU) glued to a card, labelled “Mexico: Guerrero, 6.6 mi SW Filo de Caballo, 12.vii.1985, J.B. Woolley, 85/051”. Paratypes: 10♀ on cards: COSTA RICA. **Cartago:** Cerro de la Muerte, Villa Mills, 3000m, iii-vi.1990, P. Hanson (1♀, BMNH). MEXICO. **Chiapas:** San Cristobal Reserva, Huitepec, 7700–7850’, 3.viii.1990, J.B. Woolley, 90/051B (1♀, TAMU); San Cristobal, 7200’, 25.vi.1969 (1♀, CNC); **Oaxaca**: 8mi NE El Punto, 18.vii.1985, J.B. Woolley & G. Zolnerowich, 85/074 (2♀, BMNH, TAMU); 6mi NE Mitla, 20.vii.1985, J.B. Woolley, 85/077 (1♀, BMNH); 6.8mi N Candelaria Loxicha, 3250’, 12.vii.1987, J.B. Woolley & G. Zolnerowich, 87/035 (1♀, TAMU); **Tamaulipas:** Altas Cumbre, 12mi SW Victoria, 19.iii.1986, G. Zolnerowich (1♀, TAMU); **Veracruz:** 3mi NE Huatusco, 22.vii.1985, J.B. Woolley, 85/084 (1♀, TAMU); 3.1mi NE Coscomatepec, 22.vi.1983, 3700’, R. Anderson (1♀, CNC).

##### Diagnosis.

Pronotum with a transverse carina close to posterior margin (Fig. 52); midlobe of mesoscutum with a strong median groove in posterior 1/3 (Fig. 52); scutellum with two sublateral rows of strong reticulation, remaining surface with engraved and weak reticulation to smooth and shiny (Fig. 52); postmarginal vein 0.9× as long as stigmal vein; propodeum with two submedian carinae, diverging posteriorly (Fig. 52); propodeal callus with 2–5 setae.

##### Description.

FEMALE. Length 1.2–1.8 mm.

Scape yellowish-brown to pale brown, remaining antenna dark brown. Frons below frontal suture golden-green to golden-red, above metallic bluish-purple to golden-green (Fig. 53). Vertex metallic bluish-purple to golden-green. Mesoscutum, scutellum and propodeum golden-green to metallic bluish-green (Fig. 54). Fore coxa white to dark and metallic, mid and hind coxae dark and metallic; remaining parts of legs white, except infuscate apical tarsal segment on all legs. Forewing hyaline with a weak median infuscate spot. Petiole dark brown with metallic purple tinges. First gastral tergite metallic bluish-green, remaining tergites metallic dark purple.

**Figures 50–54.**
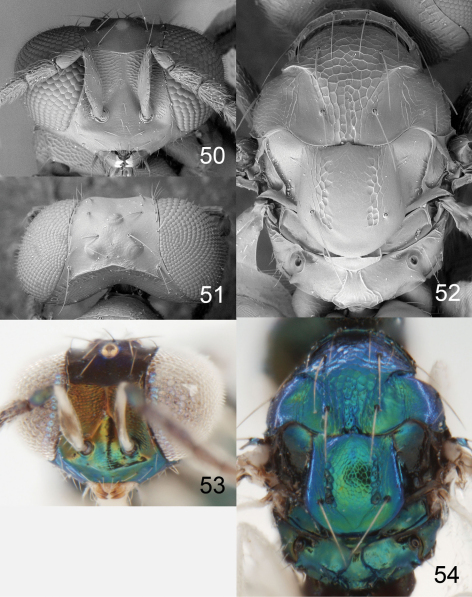
*Achrysocharoides sulcatus* sp. n., female. **50** Head, frontal **51** Vertex **52** Thoracic dorsum **53** Head, frontal **54** Thoracic dorsum.

Frons with raised and strong to weak reticulation (Fig. 50). Vertex smooth and shiny, inside ocellar triangle with very weak reticulation (Fig. 51). Occipital margin with a sharp carina behind ocellar triangle (Fig. 51). Ratios: length of flagellomeres I/II/III/IV/V (excl. spicule) 1.8/1.5/1.5/1.0/1.1.

Pronotum with a strong transverse carina close to posterior margin (Fig. 52). Midlobe of mesoscutum with raised and strong reticulation, posterior 1/3 with a strong median groove (Fig. 52); sidelobes with engraved and weak reticulation; notaular depressions smooth and shiny. Scutellum with two sublateral rows of strong reticulation, remaining surface with engraved and weak reticulation to smooth and shiny (Fig. 52). Axillae smooth and shiny (Fig. 52). Dorsellum concave to almost flat, smooth and shiny, with two foveae anterolaterally (Fig. 52). Forewing speculum closed below; costal cell in basal ½ with ventral surface with setae. Propodeum with two submedian carinae, strongly diverging posteriorly (Fig. 52); propodeal surface smooth and shiny; propodeal callus with 2–5 setae. Petiolar foramen triangular.

Petiole as long as wide to transverse, dorsal surface with weak or strong sculpture. Gaster oval-shaped.

MALE. Unknown.

##### Etymology.

From the Latin *sulcus* = groove, referring to strong groove on posteromedian mesoscutum.

##### Distribution.

Costa Rica, Mexico.

### Unplaced species

#### Achrysocharoides foveatus sp. n.

urn:lsid:zoobank.org:act:C902C8E9-E31F-4527-B24F-6616D25F5AD0

http://species-id.net/wiki/Achrysocharoides_foveatus

Figures 55–59

##### Material.

Holotype female (LUZM) glued to a card, labelled “Honduras: Francisco Morazan, Macuelizo, Tatumbla, 17.x.1995, R. Cave”.

**Diagnosis**. Pronotum without transverse carina close to posterior margin (Fig. 57); scutellum smooth with 2+3 sublateral foveae in anterior ½ (Fig. 57); postmarginal vein 1.0× as long as stigmal vein; propodeum smooth, without longitudinal carinae (Fig. 57); propodeal callus with three setae.

##### Description.

FEMALE. Length 1.2 mm.

Scape pale brown, remaining antenna dark brown. Frons below frontal suture golden-green, above golden-red (Fig. 58). Vertex metallic dark purple, golden-green inside ocellar triangle. Mesoscutum, scutellum and propodeum metallic bluish-green (Fig. 59). Coxae dark brown with metallic tinges; femora pale brown; tibiae and tarsi white. Wings hyaline. Petiole dark brown with metallic tinges. Gastral tergite 1 metallic bluish-green, remaining tergites golden-green.

**Figures 55–59.**
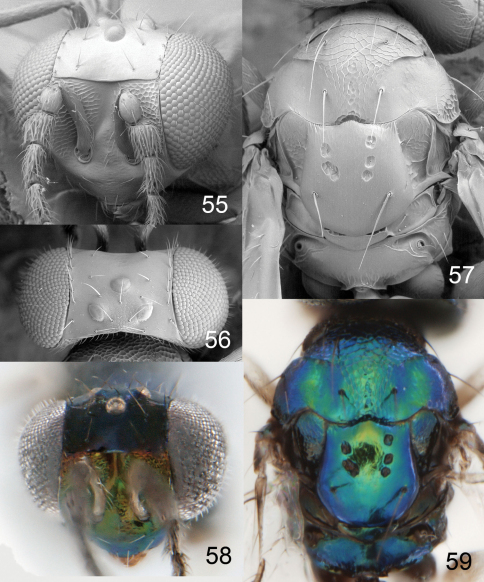
*Achrysocharoides foveatus* sp. n., female. **55** Head, frontal **56** Vertex **57** Thoracic dorsum **58** Head, frontal **59** Thoracic dorsum.

Frons with raised and strong reticulation (Fig. 55). Vertex smooth with engraved and weak reticulation inside ocellar triangle (Fig. 56). Occipital margin with sharp carina behind ocellar triangle (Fig. 56). Ratios: length of flagellomeres I/II/III/IV/V (excl. spicule) 1.4/1.6/1.6/1.2/1.0.

Pronotum without transverse carina close to posterior margin (Fig. 57). Midlobe of mesoscutum with raised and strong reticulation, sidelobes with engraved and weak reticulation (Fig. 57); notaular depressions smooth and shiny. Scutellum smooth with 2+3 sublateral foveae in anterior ½ (Fig. 57). Axillae with raised and very weak reticulation (Fig. 57). Dorsellum flat with two small foveae anterolaterally (Fig. 57). Forewing speculum closed below. Propodeum smooth, without longitudinal carinae (Fig. 57); propodeal callus with three setae. Petiolar foramen semicircular.

Petiole 0.5× as long as wide. Gaster oval-shaped.

MALE. Unknown.

##### Etymology.

From the Latin *fovea* = pit, referring to the pits on scutellum.

##### Distribution.

Honduras.

**Figures 60–63.**
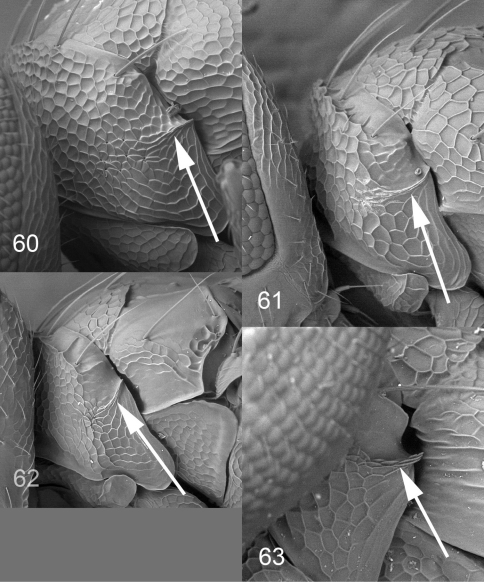
*Achrysocharoides* spp., females, lateral part of pronotum, arrow points at longitudinal carina. **60** *Achrysocharoides zwoelferi* (Delucchi), type species for *Enaysma* Delucchi **61** *Achrysocharoides usticrus* (Bouček), type species for *Kratoysma* **62** *Achrysocharoides infuscus* sp. n. **63** *Achrysocharoides ecuadorensis* (Hansson).

## Supplementary Material

XML Treatment for Achrysocharoides

XML Treatment for Achrysocharoides asperulus

XML Treatment for Achrysocharoides callisetosus

XML Treatment for Achrysocharoides cuspidatus

XML Treatment for Achrysocharoides ecuadorensis

XML Treatment for Achrysocharoides gliricidiae

XML Treatment for Achrysocharoides infuscus

XML Treatment for Achrysocharoides mediocarinatus

XML Treatment for Achrysocharoides purpureus

XML Treatment for Achrysocharoides sulcatus

XML Treatment for Achrysocharoides foveatus
